# Identification of carbapenemase genes in *Serratia *spp

**DOI:** 10.1186/1753-6561-8-S4-P39

**Published:** 2014-10-01

**Authors:** Peceu Magyve Ragagnin De Oliveira, Wirlaine Glauce Maciel, José Lourenço Dos Santos Cunha E Silva, Kesia Esther Da Silva, Maisa Estopa Correa, Nathalie Gaebler Vasconcelos, Flávia Patussi Correia Sacchi, Mariana Trinidad Ribeiro Da Costa Garcia Croda, Fábio Juliano Negrão, Júlio Henrique Rosa Croda, Simone Simionatto

**Affiliations:** 1 Faculty of Biological and Environmental Sciences, 32279804-970, Federal University of Grande Dourados, Dourados, MS, Brazil; 2 Faculty of Health Sciences, 79800-000, Federal University of Grande Dourados, Dourados, MS, Brazil

## Background

Reports of nosocomial infection due to carbapenem resistant *Serratia *spp. have become significantly more common. This resistance may be due to production of distinct carbapenemases, such as KPC [1,2]. This enzyme, initially described in *Klebsiella pneumoniae *isolates, has also been detected among other organisms, such as *Serratia marcescens*, emphasizing the global risk of interspecies spread of resistance genes [2,3]. The aim of this study was to identify carbapenemase genes in *Serratia *spp. Isolated of nosocomial infection.

## Methods

The samples were collected during May/2012 to May/2013. The strains were recovered from urinary tract, tracheal aspirate and blood culture from patients hospitalized Dourados/MS hospital. The identification of *Serratia *spp. and the sensitivity test was carried out using a Vitek (BioMérieux) automated system. All strains with reduced susceptibility to imipenem or meropenem were screened for carbapenemase production by the modified Hodge test as recommended by the Clinical and Laboratory Standards Institute [4]. The presence of KPC coding gene was assessed by PCR as described by Cuzon et al. (2010) [5].

## Results and conclusions

From May/2012 to May/2013, fifty strains of *Serratia *spp. were isolated. The wards that had the highest incidence of *Serratia *spp. were the intensive care units (ICUs). The strains identified as producing carbapenemases were evaluated by PCR using primers specific for *bla_KPC _*gene. Fourteen *Serratia *spp. strains were positive in PCR. This work describes the first report of KPC gene in *Serratia *spp. isolates in Mato Grosso do Sul, Brazil and confirm the high level of resistance of *Serratia *spp. against carbapenemics. The clinical importance of detecting carbapenemases producing *Serratia *spp. is to contribute to hospital infection control, reducing the spread of multidrug-resistant microorganisms and providing results that help in choosing the most appropriate antimicrobial, prolonging patient survival.
